# Peripheral blood CD4^+^CD25^+^CD127^low^ regulatory T cells are significantly increased by tocilizumab treatment in patients with rheumatoid arthritis: increase in regulatory T cells correlates with clinical response

**DOI:** 10.1186/s13075-015-0526-4

**Published:** 2015-01-21

**Authors:** Jun Kikuchi, Misato Hashizume, Yuko Kaneko, Keiko Yoshimoto, Naoshi Nishina, Tsutomu Takeuchi

**Affiliations:** Division of Rheumatology, Department of Internal Medicine, School of Medicine, Keio University, 35 Shinanomachi, Shinjuku-ku, Tokyo 160-8582 Japan; Product Research Department, Fuji-Gotemba Research Laboratories, Chugai Pharmaceutical Co, Ltd 1-135 Komakado, Gotemba, Shizuoka 412-8513 Japan

## Abstract

**Introduction:**

Tocilizumab (TCZ), an anti-interleukin-6 receptor antibody, is clinically effective against rheumatoid arthritis (RA), and several reports have indicated how TCZ influences a number of mechanisms underlying RA pathogenesis. However, it is still unclear whether TCZ affects inflammatory cells in peripheral blood and whether any such changes are associated with clinical response. We evaluated associations between proportions of subsets of peripheral immune cells and clinical response in patients with RA treated with TCZ.

**Methods:**

Thirty-nine consecutive patients with RA who started to receive TCZ as their first biologic between March 2010 and April 2012 were enrolled. The proportions of several subsets of peripheral cells with their levels of expression of differentiation markers, activation markers and costimulatory molecules were measured sequentially from baseline to week 52 by flow cytometry analysis.

**Results:**

Clinical Disease Activity Index (CDAI) remission was achieved in 53.8% of patients at week 52 of TCZ therapy. The proportions of CD4^+^CD25^+^CD127^low^ regulatory T cells (T_reg_) and HLA-DR^+^ activated T_reg_ cells significantly increased with TCZ therapy (*P* < 0.001 and *P* < 0.001, respectively), whereas proportions of CD3^+^CD4^+^CXCR3^−^CCR6^+^CD161^+^ T helper 17 cells did not change over the 52 weeks. The proportions of CD20^+^CD27^+^ memory B cells, HLA-DR^+^CD14^+^ and CD69^+^CD14^+^ activated monocytes, and CD16^+^CD14^+^ monocytes significantly decreased (*P* < 0.001, *P* < 0.001, *P* < 0.001 and *P* < 0.001, respectively). Among them, only the change in T_reg_ cells was inversely correlated with the change in CDAI score (ρ = −0.40, *P* = 0.011). The most dynamic increase in T_reg_ cells was observed in the CDAI remission group (*P* < 0.001).

**Conclusion:**

This study demonstrates that TCZ affected proportions of circulating immune cells in patients with RA. The proportion of T_reg_ cells among CD4^+^ cells correlated well with clinical response.

**Electronic supplementary material:**

The online version of this article (doi:10.1186/s13075-015-0526-4) contains supplementary material, which is available to authorized users.

## Introduction

T cells (especially CD4^+^ T cells), monocytes and B cells are considered to be involved in the pathogenesis of rheumatoid arthritis (RA) [1]. It is frequently considered that decreasing the number and/or activity of lymphocytes and other immune cells by RA treatment can reduce disease activity. The first evidence of this was seen in preliminary clinical trials in which targeting CD4^+^ T cells with anti-CD4 monoclonal antibodies (mAbs) resulted in clinical improvement of RA, albeit only modestly [2]. Abatacept, a cytotoxic T lymphocyte antigen 4 immunoglobulin recombinant fusion protein that inhibits CD4^+^ T cell activation by blocking costimulation with antigen-presenting cells such as B cells and monocytes, showed clinical efficacy against RA and has been approved worldwide for the treatment of RA [3]. Depletion of peripheral B cells by the anti-CD20 antibody rituximab also improves disease activity [4].

The anti-interleukin-6 receptor (IL-6R) antibody tocilizumab (TCZ) is also clinically effective against RA [5]. Several studies have shown that blocking the IL-6 signaling with TCZ can affect proportions of peripheral blood cells. Given that IL-6 was originally identified as a B cell differentiation factor [6,7], it is not surprising that TCZ affects proportions of B cell populations in patients with RA. IL-6 also influences differentiation of T cells into effector T cells (T_H_1, T_H_2 and T_H_17 cells) or regulatory T cells (T_reg_) [8-10]. In recent studies, researchers have shown that IL-6 blockade could favorably affect the T_H_17/T_reg_ cell imbalance in patients with RA [11,12]. Moreover, IL-6 seems to affect the proliferation and activation of monocytes that express IL-6R [13,14]. However, because in previous studies the number of patients, the period of study and the examined cell populations were limited, it is not clear whether there is a key population of peripheral immune cells that attenuates RA clinical symptoms through anti-IL-6R therapies [11,12,15]. If these relationships could be clarified, it would enable medical researchers to comprehend the pathogenesis of RA from the view of lymphocyte populations and to find surrogate markers in order to choose an optimal therapeutic strategy for RA.

The primary objective of this study was to evaluate multiple different types of peripheral blood cells by using flow cytometry analysis to identify populations modulated by anti-IL-6R therapy. The secondary objective was to determine whether any of these populations is strongly associated with various clinical measures in response to anti-IL-6R therapy.

## Methods

### Patients

Eligible patients were those who met the 1987 revised criteria of the American College of Rheumatology (ACR) for the classification of RA or the 2010 ACR/European League Against Rheumatism (EULAR) classification criteria [16,17]. Consecutive patients at our institute who commenced TCZ as their first biologic agent between March 2010 and April 2012 were enrolled. They all showed insufficient response to at least one conventional synthetic disease-modifying antirheumatic drug (csDMARD). The enrolled patients were administered 8 mg/kg TCZ every 4 weeks, either with or without other csDMARDs, including methotrexate (MTX). The study protocol was approved by the ethics committee at Keio University School of Medicine and was carried out in accordance with the Declaration of Helsinki and Good Clinical Practice. Written informed consent was obtained from all patients.

### Clinical assessments and evaluation of effectiveness

Demographic and clinical characteristics including age, sex, disease duration, tender joint count (TJC), swollen joint count (SJC), patient global assessment (patient visual analogue scale (Pt-VAS)), physician global assessment (doctor’s visual analogue scale (D-VAS)), Health Assessment Questionnaire Disability Index (HAQ-DI) score, C-reactive protein (CRP) level, erythrocyte sedimentation rate (ESR), matrix metalloproteinase-3 (MMP-3) level, rheumatoid factor (RF) value and anticyclic citrullinated peptide (CCP) antibody value were obtained from the patients’ medical records.

Disease activity was assessed using the Clinical Disease Activity Index (CDAI) and Simplified Disease Activity Index (SDAI). The cutoff values for remission, low disease activity (LDA), moderate disease activity (MDA) and high disease activity (HDA) were as follows: for remission, CDAI ≤2.8, SDAI ≤3.3; for LDA, 2.8 < CDAI ≤ 10, 3.3 < SDAI ≤ 11; for MDA, 10 < CDAI ≤ 22, 11 < SDAI ≤ 26; and for HDA, CDAI > 22, SDAI > 26 [18].

### Cell surface staining and flow cytometry analysis

Peripheral blood mononuclear cells (PBMCs) were obtained at baseline and at weeks 24 and 52 of TCZ treatment. PBMCs were separated by density gradient with Ficoll-Paque Plus (GE Healthcare, Uppsala, Sweden) and cryopreserved in CELLBANKER 1 (Nippon Zenyaku Kogyo, Fukushima, Japan) until use. Thawed cells were stained for 30 minutes at room temperature under darkened conditions with the following fluorophore-labeled mAbs: anti-CD4-VioGreen (Miltenyi Biotec, Bergisch Gladbach, Germany); anti-CD3-Pacific Blue/fluorescein isothiocyanate (FITC), anti-CD8-Pacific Blue, anti-CD14-(APC)-Cy7, anti-CD20 allophycocyanin-cyanine 7 (APC-Cy7), anti-CD25 phycoerythrin (PE)-Cy5, anti-CD27-PE-Cy7, anti-CD38-PE-Cy5, anti-CD45RO-PE-Cy7, anti-CD56-PE/PE-Cy7, anti-CD69-APC/PE-Cy7, anti-CD80-FITC, anti-CD86-PE-Cy5, anti-CD127-FITC, anti-CD161-APC, anti-chemokine (C-X-C motif) receptor 3 (CXCR3)-PE and anti-HLA-DR-APC/APC-Cy7 (all from BD Biosciences, Franklin Lakes, NJ, USA); anti-CD16-Brilliant Violet 510 and anti-CCR6-Brilliant Violet 421 (both from BioLegend, San Diego, CA, USA); and anti-mouse immunoglobulin G isotype-matched controls (VioGreen from Miltenyi Biotec, the others from BD Biosciences).

Stained cells were washed twice with 2 ml of phosphate-buffered saline and analyzed on a MACSQuant analyzer (Miltenyi Biotec). Dead cells were confirmed with a propidium iodide fluorescence solution (Miltenyi Biotec) and excluded on the basis of scatter signals. The subsets analyzed were CD4 and CD8 T cells (including memory, effector and activation markers) and T_H_1, T_H_2, T_H_17, T_reg_, B cells, natural killer cells, and monocytes, including their subpopulations and activation markers. The peripheral cell subsets identified in this study were defined by using cell surface markers on the basis of peripheral cell subsets described in a previous report (Additional file 1: Table S1) [19].

### Statistical analysis

Continuous data are presented as median and interquartile range (IQR) or as a number with percentage value, as appropriate. The Wilcoxon test and Kruskal-Wallis test were used to examine the differences between continuous variables. Correlation of two continuous variables was analyzed using the Spearman rank correlation coefficient. Fisher’s exact test was used to compare the proportion of categorical data between groups. A *P*-value <0.05 was considered statistically significant. All statistical analyses were performed with JMP 10 (SAS Institute, Cary, NC, USA).

## Results

### Baseline characteristics of patients and associations between peripheral cell populations and disease activity at baseline

Table 1 shows the baseline demographics and clinical characteristics of the enrolled patients (*N* = 39). In this population, there was no difference between TCZ monotherapy and TCZ in combination with MTX in terms of baseline characteristics.

**Table 1:** **Patient baseline demographics and clinical characteristics** ^**a**^

**Patient characteristics,** ***N*** **= 39**	**Mean ± SD or** ***n*** **(%)**	**Median (IQR)**
Age, yr	54.8 ± 13.3	56 (44 to 63)
Female, *n* (%)	35 (89.7)	–
Disease duration, yr	4.7 ± 3.3	4.5 (1.7 to 8.0)
SJC (range, 0 to 28)	5.8 ± 3.8	5 (3 to 7)
TJC (range, 0 to 28)	5.0 ± 3.5	4 (3 to 6)
Pt-VAS (score/100 mm)	46.2 ± 24.5	45 (30 to 63)
D-VAS (score/100 mm)	42.3 ± 16.7	39 (32 to 54)
CDAI score	19.6 ± 9.3	17.5 (12.0 to 25.2)
SDAI score	21.1 ± 9.9	19.8 (13.4 to 26.9)
HAQ-DI score	1.0 ± 0.7	1 (0.5 to 1.5)
CRP, mg/dl	1.4 ± 1.6	0.7 (0.2 to 2.2)
ESR, mm/h	48.1 ± 32.0	46 (19 to 68)
MMP-3, ng/ml	158.5 ± 147.7	100.2 (60.0 to 221.0)
RF-positive, *n* (%)	33 (84.6)	–
ACPA-positive, *n* (%)	33 (84.6)	–
Concomitant methotrexate, *n* (%), dose,^b^ mg/wk	12 (30.8), 8.0 ± 1.2	–, 8.0 (7.6 to 8.0)
Concomitant glucocorticoid, *n* (%), dose,^b^ mg/day	10 (25.6), 5.1 ± 2.8	–, 5 (3 to 5)

^a^ACPA, Anticitrullinated protein antibody; CDAI, Clinical Disease Activity Index; CRP, C-reactive protein; D-VAS, Doctor’s visual analogue scale; ESR, Erythrocyte sedimentation rate; HAQ-DI, Health Assessment Questionnaire Disability Index; MMP-3, Matrix metalloproteinase-3; Pt-VAS, Patient’s visual analogue scale; RF, Rheumatoid factor; SDAI, Simplified Disease Activity Index; SJC, Swollen joint count; TJC, Tender joint count. ^b^Mean ± standard deviation (SD) and median (interquartile range (IQR)) among patients receiving drugs.

At baseline, a higher proportion of HLA-DR^+^CD8^+^ T cells among the CD8^+^ T cells was significantly associated with higher CRP, Pt-VAS, SDAI and HAQ-DI, as well as a higher proportion of naïve and memory CD8^+^ T cells among the CD8^+^ T cells, was significantly associated with RF, ACPA, SJC, CDAI and SDAI (Additional file 1: Table S2). A higher proportion of T_H_2 cells among the CD4^+^ T cells was also significantly associated with TJC, D-VAS, Pt-VAS, CDAI and SDAI scores. No other baseline subsets or surface markers correlated with CDAI or SDAI score (Additional file 1: Table S3).

### Changes from baseline in clinical response

All patients in this study received TCZ for the entire 52 weeks. The CDAI and SDAI scores (mean ± standard deviation) significantly decreased from 19.6 ± 9.3 and 21.1 ± 9.9, respectively, at baseline to 5.5 ± 5.2 and 5.5 ± 5.2 at week 24 and to 5.2 ± 6.0 and 5.6 ± 6.8 at week 52 (*P* < 0.0001). The number and percentage of patients categorized as having attained remission or as having LDA, MDA and HDA were as follows for CDAI and SDAI: at baseline, 0 (0%) and 0 (0%) for remission, 3 (7.7%) and 4 (10.3%) for LDA, 24 (61.5%) and 25 (64.1%) for MDA, and 12 (30.8%) and 10 (25.6%) for HDA, respectively; and at week 52, 21 (53.8%) and 22 (56.4%) for remission, 11 (28.2%) and 10 (25.6%) for LDA, 7 (17.9%) and 7 (17.9%) for MDA, and 0 (0%) and 0 (0%) for HDA, respectively (Additional file 1: Figure S2).

### Changes from baseline in peripheral cell subsets

The proportions of memory CD4^+^ T cells among all CD4^+^ T cells, HLA-DR^+^CD8^+^ T cells among CD8^+^ T cells, T_reg_ cells among CD4^+^ T cells, HLA-DR^+^ T_reg_ cells among T_reg_ cells, naïve B cells among all B cells, and CD16^−^CD14^+^ monocytes among CD14^+^ monocytes increased after TCZ treatment (Tables 2 and 3). On the other hand, the proportions of naïve CD4^+^ T cells among all CD4^+^ T cells, HLA-DR^+^CD4^+^ T cells among CD4^+^ T cells, CD86^+^ B cells among all B cells, memory B cells among all B cells, HLA-DR^+^CD14^+^ monocytes among CD14^+^ monocytes, CD69^+^CD14^+^ monocytes among CD14^+^ monocytes, and CD16^+^CD14^+^ monocytes among CD14^+^ monocytes decreased after TCZ treatment (Tables 2 and 3). The other subsets and their activation markers were not significantly changed during TCZ therapy.

**Table 2:** **Changes in proportions of subsets and surface markers of T cells during tocilizumab treatment**

**Subsets and surface markers**	**Median (interquartile range)**			**Baseline vs. week 52**
	**Baseline**	**Week 24**	**Week 52**	***P-*** **value**
CD4^+^ T cells/lymphocytes	53.6 (47.3 to 59.4)	53.4 (47.3 to 58.4)	55.3 (49.5 to 60.5)	0.345
Naïve CD4^+^ T cells/CD4^+^ T cells	61.8 (57.7 to 69.3)	59.1 (53.7 to 62.2)	59.1 (51.8 to 61.8)	0.019*
Memory CD4^+^ T cells/CD4^+^ T cells	30.7 (30.7 to 42.3)	40.9 (37.8 to 46.3)	40.9 (38.2 to 48.2)	0.010*
HLA-DR^+^CD4^+^ T cells/CD4^+^ T cells	3.2 (2.7 to 4.1)	3.1 (2.6 to 3.9)	2.6 (2 to 3.1)	0.005*
CD38^+^CD4^+^ T cells/CD4^+^ T cells	32.4 (26.3 to 38.5)	32.4 (28.4 to 39.4)	32.6 (29.3 to 40.1)	0.212
CD69^+^CD4^+^ T cells/CD4^+^ T cells	0.1 (0 to 0.1)	0.1 (0 to 0.2)	0.1 (0.1 to 0.3)	0.016*
CD8^+^ T cells/lymphocytes	32.4 (28.4 to 38.1)	30.8 (26.9 to 37.1)	29.1 (26.4 to 33.1)	0.052
Naïve CD8^+^ T cells/CD8^+^ T cells	60.8 (57.7 to 64.3)	62.1 (59.5 to 67.5)	62.7 (59.5 to 67.5)	0.087
Memory CD8^+^ T cells/CD8^+^ T cells	39.2 (35.7 to 42.3)	37.9 (33.9 to 41.5)	37.3 (32.5 to 40.5)	0.087
HLA-DR^+^CD8^+^ T cells/CD8^+^ T cells	16.4 (13.4 to 19.4)	18.3 (14.3 to 19.4)	19.4 (17.3 to 21.4)	0.005*
CD38^+^CD8^+^ T cells/CD8^+^ T cells	25.2 (15.9 to 36.7)	26.1 (19.3 to 31.4)	24.5 (19.4 to 30)	0.727
CD69^+^CD8^+^ T cells/CD8^+^ T cells	1.3 (0.5 to 2.1)	1.3 (0.9 to 2.1)	0.9 (0.3 to 1.3)	0.057
T_reg_/CD4^+^ T cells	3.6 (2.2 to 4.3)	4.2 (2.6 to 5.2)	5.6 (4.5 to 7.1)	<0.001*
Naïve T_reg_/T_reg_ cells	52.8 (44.0 to 59.0)	47.7 (42.6 to 54.9)	49.6 (46.5 to 55.7)	0.242
Memory T_reg_/T_reg_ cells	47.2 (41 to 56.1)	52.3 (45.1 to 57.4)	50.4 (44.3 to 53.5)	0.242
HLA-DR^+^ T_reg_/ T_reg_ cells	14 (11.7 to 17.7)	16.8 (14.3 to 20.3)	18.4 (15.4 to 19.4)	<0.001*
T_H_1/CD4^+^ T cells	19 (14.8 to 20.8)	17.4 (15.3 to 19.3)	20.7 (15.9 to 22.9)	0.095
HLA-DR^+^ T_H_1/T_H_1 cells	4.5 (3.4 to 5.7)	4.5 (3.4 to 5.7)	3.5 (2.2 to 5.6)	0.119
T_H_2/CD4^+^ T cells	46.8 (37.7 to 54.3)	51.8 (42.5 to 58.7)	43.3 (34.6 to 49.1)	0.179
HLA-DR^+^ T_H_2/T_H_2 cells	2.0 (1.2 to 3.1)	2.3 (1.9 to 2.9)	2.2 (1.8 to 2.9)	0.174
T_H_17/CD4^+^ T cells	2.0 (0.78 to 3.2)	2.0 (1.4 to 3.2)	2.0 (1.5 to 3.6)	0.342
HLA-DR^+^ T_H_17/T_H_17 cells	4.0 (3.4 to 4.4)	3.6 (3.1 to 4.1)	3.9 (3.3 to 4.2)	0.323

*Significant differences determined using Wilcoxon’s matched-pairs signed-rank test.

**Table 3:** **Changes in proportions of subsets and surface markers of B cells, natural killer cells and monocytes during tocilizumab treatment**

**Subsets and surface markers**	**Median (interquartile range)**			**Baseline vs. week 52**
	**Baseline**	**Week 24**	**Week 52**	***P*** **-value**
B cells/lymphocytes	3.2 (2 to 5.1)	4.1 (3.1 to 5.2)	4.1 (3.1 to 5.2)	0.118
CD80^+^ B cells/B cells	26.9 (13.5 to 32)	19.3 (12.4 to 26.3)	19.3 (13.2 to 21.4)	0.045*
CD86^+^ B cells/B cells	42.5 (36.5 to 47.6)	34 (29.2 to 38.1)	36.7 (32.1 to 41.9)	0.009*
HLA-DR^+^ B cells/B cells	99.6 (99 to 99.8)	99.4 (98.8 to 99.8)	99.5 (99.3 to 99.8)	0.833
Naïve B cells/B cells	52 (43.1 to 60.3)	62.7 (52.7 to 68.2)	66.7 (57.6 to 74.6)	<0.001*
Memory B cells/B cells	48 (39.7 to 56.9)	37.3 (31.8 to 47.3)	33.3 (25.4 to 42.4)	<0.001*
NK cells/lymphocytes	24.1 (20.0 to 27.5)	25.8 (21.2 to 30.5)	24.5 (20.9 to 29.5)	0.401
CD80^+^CD14^+^ monocytes/CD14^+^ monocytes	0.2 (0.1 to 0.3)	0.2 (0.1 to 0.3)	0.2 (0.1 to 0.3)	0.433
CD86^+^CD14^+^ monocytes/CD14^+^ monocytes	99.6 (99.2 to 99.9)	99.7 (99.3 to 99.9)	99.8 (99.6 to 99.9)	0.054
HLA-DR^+^CD14^+^ monocytes/CD14^+^ monocytes	99.5 (89.3 to 99.8)	98.2 (89.7 to 99.6)	94.6 (86 to 95.8)	0.004*
CD69^+^CD14^+^ monocytes/CD14^+^ monocytes	66.7 (60.6 to 74.8)	48.3 (35.8 to 70.1)	34 (26.2 to 56.9)	<0.001*
CD16^+^CD14^+^ monocytes/CD14^+^ monocytes	16 (10.3 to 20.9)	8.6 (4.5 to 12.6)	8.1 (3.8 to 12.9)	<0.001*
CD16^−^CD14^+^ monocytes/CD14^+^ monocytes	84 (79.1 to 89.7)	91.4 (87.4 to 95.5)	91.9 (87.1 to 96.2)	<0.001*

*Significant differences using Wilcoxon’s matched-pairs signed-rank test.

### Evaluation of the relationships between peripheral cell subsets and clinical response after tocilizumab therapy

Associations between changes in peripheral cell subsets and changes in clinical endpoints (∆CDAI and ∆SDAI) after TCZ treatment are summarized in Table 4. A significant correlation was observed between the change in the proportion of T_reg_ cells among CD4^+^ T cells and the changes in CDAI score from baseline to week 52: the greater the increase in proportion of T_reg_ cells among CD4^+^ T cells from baseline to week 52, the greater the improvement in CDAI score during the same period (ρ = −0.346, *P* = 0.031). Also, the change in CD38 expression on CD8^+^ T cells over 52 weeks was negatively correlated with change in CDAI score (ρ = −0.355, *P* = 0.026). The other peripheral cell subsets, including naïve and memory CD4^+^ T cells, HLA-DR^+^CD4^+^ T cells, CD69^+^CD4^+^ T cells, HLA-DR^+^CD8^+^ T cells, HLA-DR^+^ T_reg_ naïve and memory B cells, CD86^+^ B cells, HLA-DR^+^CD14^+^ monocytes, CD69^+^CD14^+^ monocytes, CD16^+^CD14^+^ monocytes and CD16^−^CD14^+^ monocytes, the proportions of which significantly changed over 52 weeks (as shown above), did not demonstrate any correlation with the change in CDAI score. The same results were found when ΔSDAI was used as the clinical endpoint.

**Table 4:** **Correlations of changes in disease activities with changes in peripheral cell subsets and surface markers after 52 weeks of treatment with tocilizumab** ^**a**^

	**∆CDAI**		**∆SDAI**	
**Subset**	***P*** **-value**	**Spearman’s ρ**	***P*** **-value**	**Spearman’s ρ**
ΔCD4^+^ T cells/lymphocytes	0.273	0.180	0.342	0.156
ΔNaïve CD4^+^ T cells/CD4^+^ T cells	0.653	−0.074	0.481	−0.116
ΔMemory CD4^+^ T cells/CD4^+^ T cells	0.653	0.074	0.481	0.116
ΔHLA-DR^+^ CD4^+^ T cells/CD4^+^ T cells	0.829	−0.036	0.710	−0.061
ΔCD38^+^ CD4^+^ T cells/CD4^+^ T cells	0.231	−0.196	0.089	−0.276
ΔCD69^+^ CD4^+^ T cells/CD4^+^ T cells	0.746	−0.054	0.476	−0.118
ΔCD8^+^ T cells/lymphocytes	0.698	−0.064	0.294	−0.172
ΔNaïve CD8^+^ T cells/CD8^+^ T cells	0.555	0.097	0.256	0.186
ΔMemory CD8^+^ T cells/CD8^+^ T cells	0.555	−0.097	0.256	−0.186
ΔHLA-DR^+^ CD8^+^ T cells/CD8^+^ T cells	0.566	−0.095	0.955	0.009
ΔCD38^+^ CD8^+^ T cells/CD8^+^ T cells	0.026*	−0.355	0.036*	−0.337
ΔCD69^+^ CD8^+^ T cells/CD8^+^ T cells	0.686	−0.067	0.357	−0.152
ΔT_reg_/CD4^+^ T cells	0.031*	−0.346	0.033*	−0.342
ΔNaïve T_reg_/T_reg_	0.930	0.015	0.612	0.084
ΔMemory T_reg_/T_reg_	0.924	−0.016	0.606	−0.085
ΔHLA-DR^+^ T_reg_/T_reg_	0.270	−0.181	0.279	−0.178
ΔT_H_1/CD4^+^ T cells	0.847	−0.032	0.915	−0.018
ΔHLA-DR^+^ T_H_1/ T_H_1	0.969	0.007	0.732	0.057
ΔT_H_2/CD4^+^ T cells	0.354	0.153	0.312	0.166
ΔHLA-DR^+^ T_H_2/ T_H_2	0.291	−0.173	0.152	−0.234
ΔT_H_17/CD4^+^ T cells	0.593	−0.088	0.442	−0.127
ΔHLA-DR^+^ T_H_17/ T_H_17	0.366	−0.149	0.229	−0.197
ΔB cells/lymphocytes	0.958	−0.009	0.709	−0.062
ΔCD80^+^ B cells/B cells	0.740	0.055	0.546	0.100
ΔCD86^+^ B cells/B cells	0.210	0.206	0.065	0.299
ΔHLA-DR^+^ B cells/B cells	0.838	−0.034	0.841	−0.033
ΔNaïve B cells/B cells	0.291	−0.174	0.306	−0.168
ΔMemory B cells/B cells	0.291	0.174	0.306	−0.168
ΔNK cells/lymphocytes	0.559	0.097	0.506	0.110
ΔCD80^+^ CD14^+^ monocytes/CD14^+^ monocytes	0.645	−0.076	0.699	−0.064
ΔCD86^+^ CD14^+^ monocytes/CD14^+^ monocytes	0.440	0.127	0.776	0.047
ΔHLA-DR^+^ CD14^+^ monocytes/CD14^+^ monocytes	0.415	−0.134	0.138	−0.242
ΔCD69^+^ CD14^+^ monocytes/CD14^+^ monocytes	0.430	−0.130	0.258	−0.186
ΔCD16^+^CD14^+^ monocytes/CD14^+^ monocytes	0.120	−0.253	0.203	−0.208
ΔCD16^−^CD14^+^ monocytes/CD14^+^ monocytes	0.121	0.253	0.204	0.208

^a^CDAI, Clinical Disease Activity Index; HLA, Human leukocyte antigen; NK, Natural killer; SDAI, Simplified Disease Activity Index; T_H_, Helper T cell; T_reg_, Regulatory T cell. *Significant correlation using Spearman’s rank correlation coefficient.

### Association between the change in T_reg_ cells and the effectiveness of tocilizumab therapy

**Figure 1:**
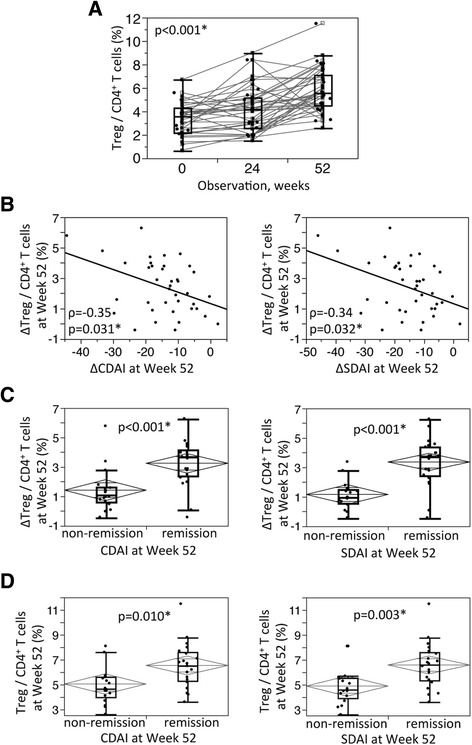
**Relations between effects of tocilizumab on the proportion of regulatory T cells and clinical responses. (A)** Chronological change in the proportion of regulatory T (T_reg_) cells among CD4^+^ T cells from baseline to week 52 of tocilizumab (TCZ) treatment. **(B)** The relationships between the change in clinical activities (Clinical Disease Activity Index (ΔCDAI) and Simplified Disease Activity Index (ΔSDAI)) and the change in proportion of T_reg_ cells among CD4+ T cells (ΔT_reg_/CD4) at week 52. **(C)** The relationships between CDAI and SDAI scores at week 52 and the change in proportion of T_reg_ cells among CD4^+^ T cells (ΔT_reg_/CD4) at week 52. **(D)** The relationships between CDAI and SDAI scores at week 52 and the proportion of T_reg_ cells among CD4^+^ T cells at week 52. The squares signify medians and interquartile ranges, and the diamonds signify means and 95% confidence intervals. Data were analyzed by using the Kruskal-Wallis test **(A)**, Spearman’s rank correlation coefficient **(B)** and the Wilcoxon rank-sum test **(C,D)**. *Significant differences.

The time course of changes in T_reg_ cells as a proportion of CD4^+^ T cells is shown in Figure 1A. The median proportion of T_reg_ cells significantly increased over 52 weeks from 3.6 (IQR, 2.2 to 4.3) to 5.6 (IQR 4.5 to 7.1) (*P* < 0.001). The change in CDAI and SDAI scores at week 52 was significantly associated with the change in T_reg_ cells at week 52 (Table 4, Figure 1B). Of the 39 patients examined in this study, 21 (53.8%) met the CDAI criteria for remission and 22 (56.4%) attained SDAI remission at week 52. When we divided the patients into two groups according to their remission status at week 52, the change in peripheral blood T_reg_ cells was significantly higher in the remission group than in the nonremission group at week 52 (Figure 1C). Moreover, the proportion of peripheral blood T_reg_ cells was significantly higher in the remission group than in the nonremission group at week 52 (Figure 1D).

As for CD38 expression on CD8^+^ T cells, the overall expression did not change during 52 weeks (*P* = 0.913), and there was no difference in its proportion between the remission and nonremission groups at Week 52 (*P* = 0.453 for CDAI, *P* = 0.345 for SDAI).

## Discussion

In this article, we report the results of the first comprehensive study to show the effect of TCZ on various peripheral cell subsets. In this study, we have shown that the proportion of CD4^+^CD25^+^CD127^low^ T_reg_ cells among CD4^+^ cells and the proportion of HLA-DR^+^-activated T_reg_ cells among T_reg_ cells significantly increased from baseline over the course of treatment with TCZ, and also that the proportions of CD20^+^CD27^+^ memory B cells, HLA-DR^+^CD14^+^ activated monocytes, CD69^+^CD14^+^ activated monocytes, and CD16^+^CD14^+^ nonclassical monocytes significantly decreased from baseline. Among them, only the increase in T_reg_ cells was significantly associated with achieving remission by TCZ treatment.

Related studies have reported the increase in T_reg_ cell levels 1 year after TCZ administration in a small population [20], for 3 months [15] and for 6 months [12]. Though definitions of T_reg_ cells in our study, using only cell surface markers without stimulatory modification for cells *in vitro*, were different from those in these previous studies, our results showed the same trends as previously reported. The mechanism is a question that is not yet fully understood. However, this is still an important biomarker, as no suitable biomarker that can help predict outcome of RA has not been identified.

Several mechanisms may contribute to the potentiation of T_reg_ cells by TCZ therapy. It is conceivable that IL-6 has an effect on T_reg_ differentiation via inhibiting the expression of a specific transcriptional factor, FoxP3 [21,22]. This may suggest a mechanism by which neutralizing of IL-6 signaling in patients with RA can induce an increase in the number and function of T_reg_ cells. The quantitative increase in T_reg_ cells expressing elevated levels of human leukocyte antigen (HLA)-DR in patients with RA treated with TCZ could also be a result of activation and proliferation of preexisting T_reg_ cells or of their differentiation by conversion from Foxp3^−^ precursors.

One question arising from the result is whether the increase of T_reg_ proportion after TCZ therapy is a result of disease remission or is attributable to use of TCZ. To answer the question, we also analyzed peripheral T_reg_ cells in 12 patients with RA longitudinally treated with MTX alone (Additional file 1: Table S4). All the patients achieved LDA or remission at week 52 after starting MTX therapy (Additional file 1: Figure S3). The proportion of T_reg_ cells did not show a certain tendency or change over 52 weeks after administration of MTX (*P* = 0.729) (Additional file 1: Figure S4A). In addition, the change in T_reg_ cells at week 52 was not associated with the change in CDAI and SDAI scores at week 52 (Additional file 1: Figure S4B). These results suggest that, albeit the comparison in a small number of patients with MTX, the increase in T_reg_ proportion after TCZ therapy is not a result of disease remission but caused by TCZ therapy itself.

Another question is whether other biologic agents for RA treatment also induce T_reg_ cells. It has been reported that neither adalimumab, an anti-tumor necrosis factor (anti-TNF) mAb, nor etanercept, a soluble TNF receptor, modified percentages or absolute numbers of circulating CD4^+^CD25^high^ T_reg_ cells or other T_reg_ phenotypes after being administered for 6 and 12 weeks to patients with RA, regardless of their response [23]. However, there is one report that high concentrations of TNFα can block the immunosuppressive functions of T_reg_ cells *in vitro* and that the treatment of patients with RA with infliximab, an anti-TNF mAb, bolsters T_reg_ suppression of the proliferation of effector cells [24]. It is assumed that these conflicting results might be ascribed to a small number of subjects (*N* = 10 to 30) and a short observational period (12 to 24 weeks). Regarding abatacept, the therapy diminishes the absolute numbers of T_reg_ cells but enhances their function in patients with RA [25]. It is conceivable that the different targets of these therapies influence the different performance of T_reg_ cells and that the results of the present study indicate a part of the unique mechanism of TCZ.

Although much still remains to be clarified about how T_reg_ defects might contribute to the pathogenesis of RA, approaches that specifically boost T_reg_ activity could be useful in the treatment of RA. In this study, the change in T_reg_ was correlated with disease activities after TCZ therapy. This is the first time that the effect of increasing T_reg_ has been shown to be strictly associated with clinical efficacy.

The effects of IL-6 on the late stages of B cell differentiation *in vitro* are well documented [26]. *In vivo* IL-6 overexpression is associated with B cell hyperactivity, autoantibody production and immunopathology [27,28]. In patients with RA, chronic activation of B cells and an accumulation of memory B cells in the peripheral blood and synovial membranes have been described [29,30]. Within this context, B cell–targeted therapies utilizing rituximab have been widely explored in RA. Because IL-6 has been described as an important B cell–stimulating factor with effects on memory B cell survival and on plasma cell differentiation and survival in the bone marrow, it is easy to comprehend the effect of TCZ on peripheral B cells, especially the ratio of naïve B cells to memory B cells [31]. Although the proportion of memory B cells significantly decreased over 52 weeks of TCZ therapy, it did not correlate with any component of activity status, SJC, TJC, Pt-VAS, D-VAS, CRP and ESR. Therefore, the decrease can be attributed to the effect of TCZ therapy rather than to disease activity. When we compared the proportion of B cell subsets in the same 12 patients that were effectively treated with MTX alone during 52 weeks as mentioned above (Additional file 1: Table S5), we observed that the proportion of memory B cells tended to decrease in patients with MTX therapy, as in the case with TCZ, suggesting that the trend was not specific to TCZ therapy. However, the proportions of CD80^+^ and CD86^+^ B cells among all B cells did not change in patients who received MTX therapy. Therefore, the decrease in the proportion after TCZ therapy may be characteristic of TCZ.

In peripheral blood, two monocyte subpopulations with distinct functional properties have been defined by their expression of CD14 and CD16 molecules. Compared with classical CD14^+^CD16^−^ monocytes, CD16^+^ nonclassical monocytes have been shown to possess several features of inflammatory tissue macrophages, notably, higher expression of major histocompatibility complex class II antigens and several adhesion molecules and lower expression of IL-10, transforming growth factor β, macrophage colony-stimulating factor, IL-1β and TNFα [32]. The pathophysiologic significance of the CD16^+^ nonclassical monocyte subset has been demonstrated by its expansion under various inflammatory conditions, such as RA, sepsis, asthma and solid tumors. We demonstrated that TCZ reduced the peripheral level of CD16^+^ nonclassical monocytes. Although it remains to be clarified how monocytes differentiate into CD16^+^ nonclassical monocytes, this study revealed that IL-6 appears to be involved in the proliferation of CD16^+^ nonclassical monocytes or in shifting the balance of monocytes to CD16^+^ nonclassical monocytes. In the same 12 patients with RA described above who were treated with MTX alone, the proportions of HLA-DR^+^CD14^+^ monocytes and CD69^+^CD14^+^ monocytes among all monocytes did not change (Additional file 1: Table S5), which was different from the results for TCZ. The decrease of those activated monocytes after TCZ therapy did not seem to be the cause of disease remission, but rather the effect of TCZ. The proportion of CD16^+^CD14^+^ nonclassical monocytes tended to decrease during 52 weeks of MTX therapy compared with that of TCZ therapy. This may have been a result of the improvement of RA disease activity, or there may some actions on immune cells in common between TCZ and MTX.

## Conclusions

Our findings suggest that TCZ affected proportions of circulating T_reg_ cells, B cells and monocytes in patients with RA. Especially, the increase in the proportion of T_reg_ cells among CD4^+^ T cells correlated well with clinical response. Then the possible mode of action of TCZ against RA could increase the proportion of T_reg_ cells.

## Additional file

Additional file 1: Table S1. Identified subsets of peripheral blood mononuclear cells defined using cell surface markers. **Table S2.** Correlations between peripheral T cell subsets and serum markers, composite measures of disease activity, and Health Assessment Questionnaire Disability Index score at baseline. Spearman’s rank correlation coefficient was used for analysis. **Table S3.** Correlations between peripheral B cell subsets, natural killer (NK) cells and monocyte subsets and serum markers, composite measures of disease activity, and Health Assessment Questionnaire Disability Index score at baseline. Spearman’s rank correlation coefficient was used for analysis. **Table S4.** Baseline demographics and clinical characteristics of the patients treated with MTX. **Table S5.** Changes in proportions (%) of subsets and surface markers of B cells and monocytes during MTX treatment. Subsets and surface markers were selected as the changed ones in TCZ therapy. **Figure S1.** Gating strategy for flow cytometry analysis of T_reg_ cells and subsets of monocytes. **Figure S2.** Chronological changes in and proportions of clinical activity from baseline until week 52 of TCZ treatment. **Figure S3.** Chronological changes in clinical activity from baseline until week 52 of MTX treatment. **Figure S4.** Relationships between effects of MTX on the proportion of T_reg_ cells and clinical responses. **(A)** Chronological change in the proportion of T_reg_ cells among CD4^+^ T cells from baseline to week 52 of MTX treatment. **(B)** The relationships between the change in clinical activity (ΔCDAI and ΔSDAI scores) and the change in proportion of T_reg_ cells among CD4+ T cells (ΔT_reg_/CD4) at week 52. The squares signify medians and interquartile ranges.

## Abbreviations

ACPA: Anticitrullinated protein antibody
ACR: American College of Rheumatology
CCP: Cyclic citrullinated peptide
CDAI: Clinical Disease Activity Index
CRP: C-reactive protein
csDMARD: Conventional synthetic disease-modifying antirheumatic drug
D-VAS: Doctor’s visual analogue scale
ESR: Erythrocyte sedimentation rate
EULAR: European League Against Rheumatism
HAQ-DI: Health Assessment Questionnaire Disability Index
HDA: High disease activity
IL-6R: Interleukin-6 receptor
IQR: Interquartile range
LDA: Low disease activity
mAb: Monoclonal antibody
MDA: Moderate disease activity
MMP-3: Matrix metalloproteinase-3
MTX: Methotrexate
PBMC: Peripheral blood mononuclear cell
Pt-VAS: Patient’s visual analogue scale
RA: Rheumatoid arthritis
RF: Rheumatoid factor
SDAI: Simplified Disease Activity Index
SJC: Swollen joint count
TCZ: Tocilizumab
TJC: Tender joint count
TNF: Tumor necrosis factor
Treg: Regulatory T cell
