# Supplementary material for: Peripheral blood CD4+CD25+CD127low regulatory T cells are significantly increased by tocilizumab treatment in patients with rheumatoid arthritis: increase in regulatory T cells correlates with clinical response
Source: Arthritis Res Ther. 2015 Jan 21;17(1):10. doi: 10.1186/s13075-015-0526-4 (PMC4332922; doi:10.1186/s13075-015-0526-4)
Supplement: Additional file 1: Table S1. — Identified subsets of peripheral blood mononuclear cells defined using cell surface markers. Table S2. Correlations between peripheral T cell subsets and serum markers, composite measures of disease activity, and Health Assessment Questionnaire Disability Index score at baseline. Spearman’s rank correlation coefficient was used for analysis. Table S3. Correlations between peripheral B cell subsets, natural killer (NK) cells and monocyte subsets and serum markers, composite measures of disease activity, and Health Assessment Questionnaire Disability Index score at baseline. Spearman’s rank correlation coefficient was used for analysis. Table S4. Baseline demographics and clinical characteristics of the patients treated with MTX. Table S5. Changes in proportions (%) of subsets and surface markers of B cells and monocytes during MTX treatment. Subsets and surface markers were selected as the changed ones in TCZ therapy. Figure S1. Gating strategy for flow cytometry analysis of Treg cells and subsets of monocytes. Figure S2. Chronological changes in and proportions of clinical activity from baseline until week 52 of TCZ treatment. Figure S3. Chronological changes in clinical activity from baseline until week 52 of MTX treatment. Figure S4. Relationships between effects of MTX on the proportion of Treg cells and clinical responses. (A) Chronological change in the proportion of Treg cells among CD4+ T cells from baseline to week 52 of MTX treatment. (B) The relationships between the change in clinical activity (ΔCDAI and ΔSDAI scores) and the change in proportion of Treg cells among CD4+ T cells (ΔTreg/CD4) at week 52. The squares signify medians and interquartile ranges. [file 13075_2015_526_MOESM1_ESM.pdf]

Additional file 1

Table S1. Identified subsets of peripheral blood mononuclear cells defined using cell-surface markers

Identified subsets	Definition by cell-surface markers
$CD4^+$ T cells	$CD3^+CD4^+$
Naïve $CD4^+$ T cells	$CD3^+CD4^+CD45RO^-$
Memory $CD4^+$ T cells	$CD3^+CD4^+CD45RO^+$
T_{reg} cells	$CD3^+CD4^+CD25^+CD127^{low}$
T_h1 cells	$CD3^+CD4^+CXCR3^+CCR6^-$
T_h2 cells	$CD3^+CD4^+CXCR3^-CCR6^-$
T_h17 cells	$CD3^+CD4^+CXCR3^-CCR6^+CD161^+$
$CD8^+$ T cells	$CD3^+CD8^+$
Naïve $CD8^+$ T cells	$CD3^+CD8^+CD45RO^-$
Memory $CD8^+$ T cells	$CD3^+CD8^+CD45RO^+$
B cells	$CD3^-CD20^+$
Naïve B cells	$CD3^-CD20^+CD27^-$
Memory B cells	$CD3^-CD20^+CD27^+$
Natural Killer cells	$CD3^-CD56^+$
Non-classical monocytes	$CD3^-CD14^+CD16^+$
Classical monocytes	$CD3^-CD14^+CD16^-$

Table S2. Correlations between peripheral T cell subsets and serum markers, composite measures of disease activity, and Health Assessment Questionnaire-Disability Index score at baseline. Spearman rank correlation coefficient was used for analysis.

	RF	ACPA	CRP	TJC	SJC	D-VAS	Pt-VAS	HAQ-DI	CDAI	SDAI
**$CD4^+$ T cells / lymphocytes**	−0.14	−0.22	0.07	−0.08	0.08	0.00	−0.10	−0.08	0.00	0.01
**Naïve $CD4^+$ T cells / $CD4^+$ T cells**	−0.07	0.11	−0.23	−0.05	0.01	−0.07	−0.27	0.03	−0.11	−0.15
**Memory $CD4^+$ T cells / $CD4^+$ T cells**	0.07	−0.11	0.23	0.05	−0.01	0.07	0.27	−0.03	0.11	0.15
**$HLA\text{-}DR^+CD4^+$ T cells / $CD4^+$ T cells**	−0.25	−0.08	0.17	0.04	0.15	0.15	0.17	−0.05	0.23	0.24
**$CD38^+CD4^+$ T cells / $CD4^+$ T cells**	−0.10	−0.07	−0.27	−0.09	−0.08	−0.13	−0.17	−0.15	−0.17	−0.20
**$CD69^+CD4^+$ T cells / $CD4^+$ T cells**	−0.03	0.28	−0.09	−0.07	−0.02	−0.11	−0.29	−0.23	−0.13	−0.14
**$CD8^+$ T cells / lymphocytes**	0.15	−0.01	−0.04	0.08	0.21	0.15	0.05	0.21	0.16	0.15
**Naïve $CD8^+$ T cells / $CD8^+$ T cells**	0.35*	0.31*	0.14	0.26	0.34*	0.22	0.18	0.25	0.37*	0.33*
**Memory $CD8^+$ T cells / $CD8^+$ T cells**	−0.35*	−0.31*	−0.14	−0.26	−0.34*	−0.22	−0.18	−0.25	−0.37*	−0.33*
**$HLA\text{-}DR^+CD8^+$ T cells / $CD8^+$ T cells**	0.00	−0.07	0.41*	0.12	0.19	0.18	0.34*	0.30*	0.29	0.34*
**$CD38^+CD8^+$ T cells / $CD8^+$ T cells**	−0.07	−0.15	0.23	0.10	0.07	−0.08	0.20	−0.11	0.08	0.12
**$CD69^+CD8^+$ T cells / $CD8^+$ T cells**	0.07	0.00	−0.22	0.00	−0.24	−0.11	−0.11	−0.07	−0.19	−0.20
**T_{reg} / $CD4^+$ T cells**	0.09	−0.07	0.07	0.21	0.30*	−0.09	0.05	0.00	0.15	0.11
**Naïve T_{reg} / T_{reg}**	0.06	−0.01	0.42*	−0.11	0.12	0.09	0.14	−0.07	0.10	0.16
**Memory T_{reg} / T_{reg}**	−0.06	0.01	−0.42*	0.11	−0.12	−0.09	−0.14	0.07	−0.10	−0.16
**$HLA\text{-}DR^+$ T_{reg} / T_{reg}**	0.21	−0.09	0.08	0.08	−0.15	0.16	0.37*	0.17	0.10	0.10
**T_h1 / $CD4^+$ T cells**	0.21	0.04	0.06	0.09	0.09	0.17	−0.04	0.09	0.09	0.05
**$HLA\text{-}DR^+$ T_h1 / T_h1**	−0.11	0.26	0.11	-0.25	0.03	−0.05	−0.28	−0.17	−0.15	−0.14
**T_h2 / $CD4^+$ T cells**	0.31	0.12	0.11	0.37*	0.24	0.32*	0.35*	0.29	0.37*	0.36*
**$HLA\text{-}DR^+$ T_h2 / T_h2**	−0.18	0.00	−0.28	−0.19	−0.22	−0.10	−0.05	−0.12	−0.22	−0.23
**T_h17 / $CD4^+$ T cells**	0.08	0.00	−0.22	0.00	−0.23	−0.10	−0.11	−0.07	−0.19	−0.19
**$HLA\text{-}DR^+$ T_h17 / T_h17**	0.05	−0.21	−0.14	0.07	0.24	0.10	−0.04	−0.31	0.11	0.07

*P<0.05

ACPA, anti-citrullinated protein antibody; CRP, C-reactive protein; HAQ-DI, Health Assessment Questionnaire Disability Index; RF, rheumatoid factor; SJC, swollen joint count; TJC, tender joint count.

Table S3. Correlations between peripheral B cell subsets, NK cells and monocyte subsets and serum markers, composite measures of disease activity, and Health Assessment Questionnaire-Disability Index score at baseline. Spearman rank correlation coefficient was used for analysis.

	RF	ACPA	CRP	TJC	SJC	D-VAS	Pt-VAS	HAQ-DI	CDAI	SDAI
**B cells / lymphocytes**	−0.01	0.02	0.16	−0.14	0.09	0.03	−0.02	0.11	−0.04	−0.04
**$CD80^+$ B cells / B cells**	−0.01	0.04	0.01	0.17	0.14	0.06	0.09	0.00	0.15	0.12
**$CD86^+$ B cells / B cells**	0.08	0.22	0.24	−0.09	−0.06	−0.02	−0.02	0.11	−0.07	−0.02
**HLA-DR^+ B cells / B cells**	−0.25	0.05	−0.16	−0.09	0.01	−0.07	−0.16	−0.14	−0.11	−0.13
**Naïve B cells / B cells**	0.22	0.26	0.43*	0.22	0.11	−0.01	0.23	0.28	0.20	0.22
**Memory B cells / B cells**	−0.22	−0.26	−0.43*	−0.22	−0.11	0.01	−0.23	−0.28	−0.20	−0.22
**NK cells / lymphocytes**	-0.21	-0.01	-0.10	-0.23	-0.30	-0.21	-0.06	-0.12	-0.24	-0.25
**$CD80^+$ $CD14^+$ monocytes / $CD14^+$ monocytes**	−0.15	0.06	−0.06	0.04	−0.20	−0.22	−0.12	−0.24	−0.18	−0.18
**$CD86^+$ $CD14^+$ monocytes / $CD14^+$ monocytes**	−0.01	0.10	−0.09	0.37	0.23	0.01	0.14	−0.01	0.27	0.21
**HLA-DR^+ $CD14^+$ monocytes / $CD14^+$ monocytes**	0.09	0.05	−0.11	0.13	−0.06	0.11	0.22	0.06	0.10	0.08
**$CD69^+$ $CD14^+$ monocytes / $CD14^+$ monocytes**	−0.16	−0.06	0.01	−0.14	−0.28	−0.23	−0.30*	−0.17	−0.27	−0.26
**$CD16^+$ $CD14^+$ monocytes / $CD14^+$ monocytes**	0.24	0.20	0.17	−0.01	0.03	0.06	0.17	0.37*	0.06	0.11
**$CD16^-$ $CD14^+$ monocytes / $CD14^+$ monocytes**	−0.25	−0.20	−0.17	0.01	−0.03	−0.06	−0.17	−0.37*	−0.06	−0.11

*$P<0.05$

ACPA, anti-citrullinated protein antibody; CRP, C-reactive protein; HAQ-DI, Health Assessment Questionnaire Disability Index; RF, rheumatoid factor; SJC, swollen joint count; TJC, tender joint count.

Figure S1. Gating strategy for flowcytometry analysis of T_{reg} and subsets of monocytes

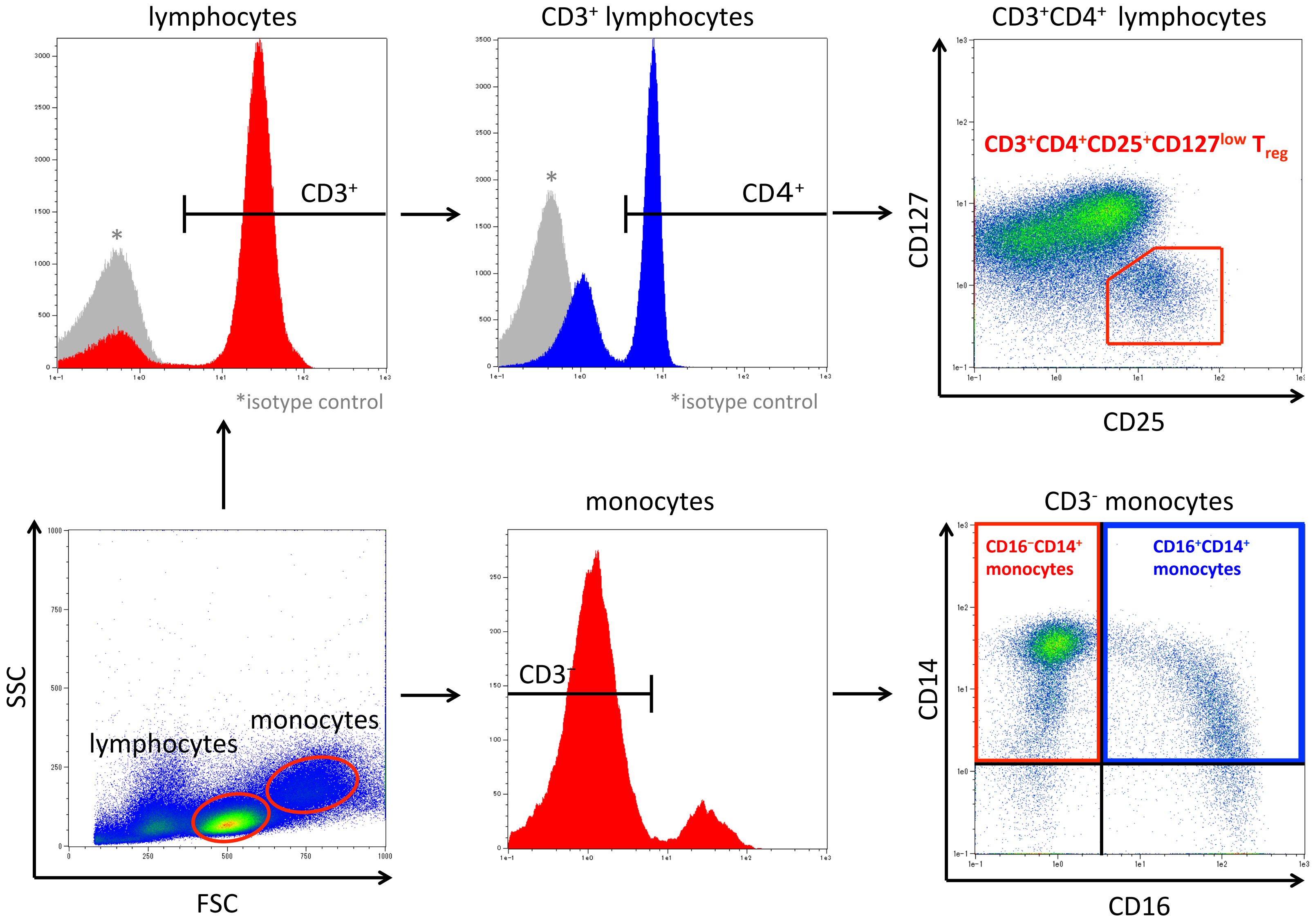

Figure S2. Chronological changes in and proportion of clinical activity from baseline until Week 52 of TCZ treatment. R, remission; L, low disease activity; M, moderate disease activity; H, high disease activity; CDAI, Clinical Disease Activity Index; SDAI, Simplified Disease Activity Index.

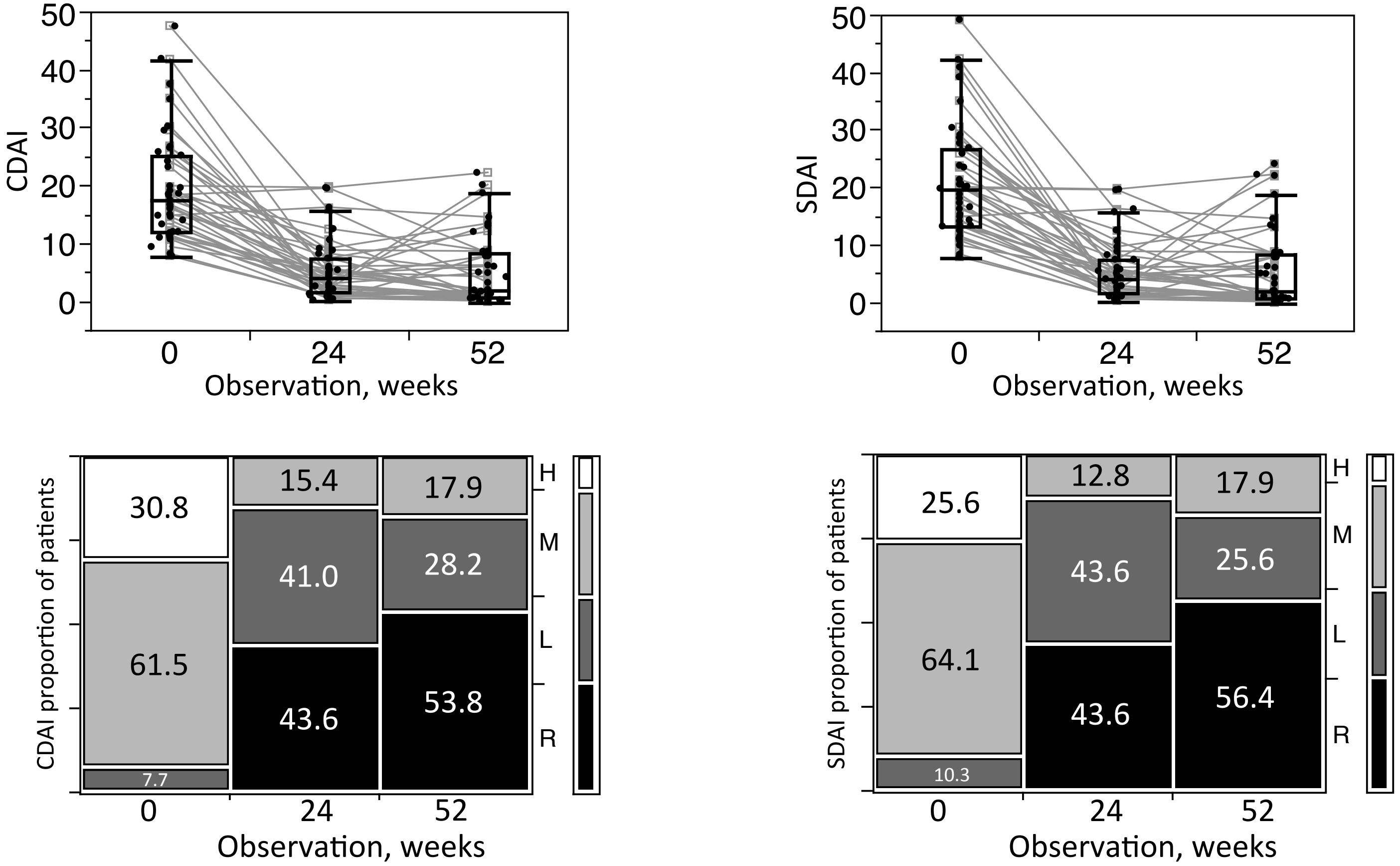

Table S4. Baseline demographics and clinical characteristics of the patients treated with MTX

Patient Characteristics, *n*=12	Mean ± SD or Number (%)	Median (IQR)
Age, years	57.6 ± 12.5	58.0 (45.0–71.0)
Female, n (%)	9 (75.0)	–
Disease duration, years	25.7 ± 45.4	11 (2.3–29.3)
Dose of methotrexate, mg/week	7.3 ± 1.0	8.0 (6.0–8.0)
Concomitant glucocorticoid, n (%)	0 (0)	–
SDAI	29.6 ± 12.1	23.7 (21.6–39.5)
CDAI	28.6 ± 11.5	23.1 (21.4–38.1)
RF positive, n (%)	8 (66.7)	–
ACPA positive, n (%)	6 (50.0)	–
CRP, mg/dl	0.98 ± 0.92	0.7 (0.2–1.9)
ESR, mm/h	52.0 ± 32.7	45.5 (25.8–76.8)
MMP-3, ng/ml	112.7 ± 112.6	69.5 (43.3–162.8)
HAQ-DI	0.7 ± 0.8	0.63 (0.1–1.2)

ACPA, anti-citrullinated protein antibody; CDAI, Clinical Disease Activity Index; CRP, C-reactive protein; ESR, erythrocyte sedimentation rate; HAQ-DI, Health Assessment Questionnaire Disability Index score; MMP-3, matrix metalloproteinase 3; MTX, methotrexate; RF, rheumatoid factor; SDAI, simplified disease activity index

Figure S3. Chronological changes in clinical activity from baseline until Week 52 of MTX treatment. CDAI, Clinical Disease Activity Index; SDAI, Simplified Disease Activity Index.

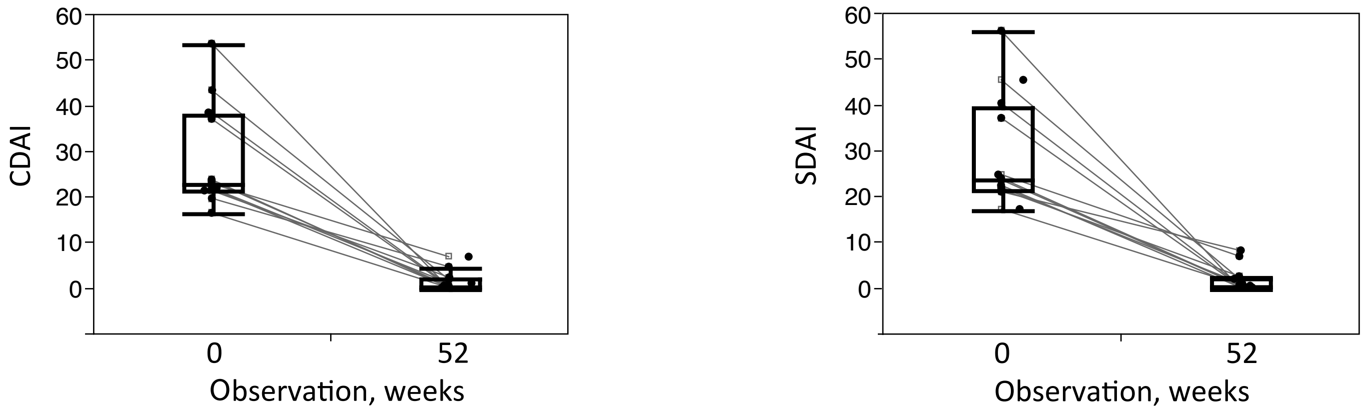

Figure S4. Relations between effects of MTX on the proportion of T_{reg} and clinical responses. **(A)** Chronological change in the proportion of T_{reg} among $CD4^{+}$ T cells from baseline to Week 52 of MTX treatment. **(B)** The relations between the change in clinical activities (ΔCDAI and ΔSDAI) and the change in proportion of T_{reg} among CD4+ T cells (ΔT_{reg} / CD4) at Week 52. The squares signify medians and interquartile ranges.

Data were analyzed by Kruskal-Wallis's test in **A**; Spearman's rank correlation coefficient in **B.**

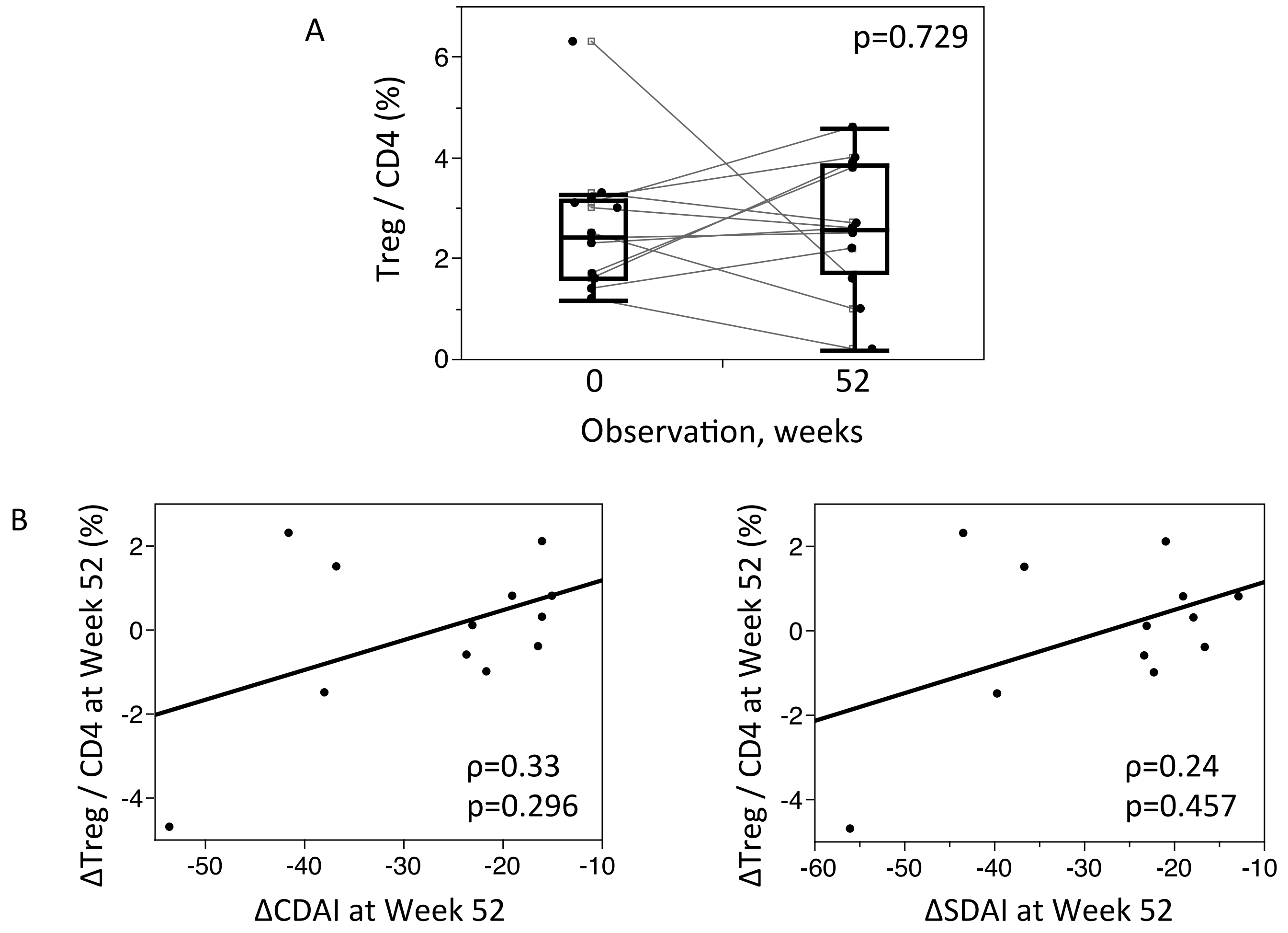

Table S5. Changes in proportions (%) of subsets and surface markers of B cells and monocytes during MTX treatment. Subsets and surface markers were selected as the changed ones in TCZ therapy.

**Subsets and surface markers**	**Median (IQR)**		**Baseline vs. Week 52**
	**Baseline**	**Week 52**	***P*-value**
$CD80^+$ B cells / B cells	10.6 (6.3–13.6)	9.4 (5.3–14.3)	0.817
$CD86^+$ B cells / B cells	74.3 (63.7–84.7)	75.8 (60.5–78.6)	0.729
Naïve B cells / B cells	65.6 (63.5–74.0)	74.8 (72.1–84.0)	0.073
Memory B cells / B cells	34.5 (27.1–36.5)	25.2 (15.8–28.3)	0.065
$HLA\text{-}DR^+$ $CD14^+$ monocytes / $CD14^+$ monocytes	67.1 (57.6–75.2)	66.2 (61.5–72.8)	0.921
$CD69^+$ $CD14^+$ monocytes / $CD14^+$ monocytes	92.4 (87.5–94.5)	94.8 (93.0–96.0)	0.106
$CD16^+CD14^+$ monocytes / $CD14^+$ monocytes	26.4 (17.6–33.2)	16.9 (8.7–27.7)	0.057
$CD16^-CD14^+$ monocytes / $CD14^+$ monocytes	73.6 (66.8–82.4)	83.1 (72.3–91.3)	0.057
